# Spinal correction of scoliosis in Jeune syndrome: a report of two cases

**DOI:** 10.1186/s13013-016-0069-8

**Published:** 2016-03-02

**Authors:** Wataru Saito, Gen Inoue, Takayuki Imura, Toshiyuki Nakazawa, Masayuki Miyagi, Takanori Namba, Eiki Shirasawa, Naonobu Takahira, Masashi Takaso

**Affiliations:** Department of Orthopaedic Surgery, Kitasato University School of Medicine, 1-15-1, Kitazato, Minami, Sagamihara, Kanagawa 252-0374 Japan; Department of Rehabilitation, Kitasato University School of Allied Health Sciences, Sagamihara, Kanagawa Japan

**Keywords:** Jeune syndrome, Asphyxiating thoracic dystrophy, Scoliosis, Spinal correction

## Abstract

**Background:**

Jeune syndrome (asphyxiating thoracic dystrophy) is an autosomal recessive disorder with constriction and narrowing of the thorax. To our knowledge, there are no reports regarding spinal deformity and correction in Jeune syndrome. Herein, we report two cases of spinal correction in patients with Jeune syndrome, and review the literature.

**Case Presentation:**

We experienced cases of spinal scoliosis in an adolescent boy and a young adult woman, both with Jeune syndrome. Their spinal deformities had progressed by the time they came to our hospital for surgical correction. After preoperative evaluation of their general condition, including respiratory function in detail to confirm that they could undergo surgery, we treated both cases with posterior spinal correction and fusion. Spinal correction was performed safely and there were no severe complications, including respiratory depression, associated with surgery, and relatively satisfactory correction was obtained in both cases. In case 1, coronal deformity was corrected from 70° to 36° and from 82° to 42°, respectively. In case 2, Cobb angle was corrected from 52° to 20° and from 55° to 21°.

**Conclusions:**

Posterior spinal correction can be performed safely in young patients with Jeune syndrome who have survived their infant stage and matured without a severe general condition.

## Background

Jeune syndrome was originally described as an asphyxiating thoracic dystrophy [1, 2]. We now know that Jeune syndrome is a rare autosomal recessive osteochondrodysplasia with characteristic skeletal abnormalities including a small and narrow chest with some respiratory complications, limb shortening and short squared iliac wings [3]. Patients surviving infancy may develop progressive renal failure in their later life [4].

There are a few reports or comments in the literature regarding the association between Jeune syndrome and spinal disorders [3, 5–7]. However, to our knowledge, there are no detailed reports of the treatment of scoliosis in this syndrome. We experienced two patients who underwent spinal correction for Jeune syndrome after surviving their infancy. We performed posterior spinal correction and found no perioperative complications following a similar strategy to that used for adolescent idiopathic scoliosis (AIS) surgery. This is the first report that presents posterior spinal correction of scoliosis in Jeune syndrome. In this report, we describe the clinical course of two surgical cases in detail and classify these cases clinically. We discuss the characteristics of spinal deformity in these cases compared with AIS.

## Case presentation

### Case 1

#### History

A 17-year-old adolescent boy with Jeune syndrome underwent spinal correction. He was born abroad and immigrated to Japan when he was 5 months old. He survived infancy, although he was diagnosed with Jeune syndrome as the cause of thoracic deformity, renal dysfunction, and urinary tract abnormality at 11 months old at a university hospital. His renal condition became worse with age. He underwent a renal transplant to treat chronic renal failure at 10 years old. Although it was not clear when his spinal deformity was discovered, he had received no treatment until after he entered junior high school. He was treated with a brace at 14 years old. However, he disliked the brace and discontinued using it within a few months. His spinal deformity progressed and he was referred to our university hospital to treat scoliosis at the age of 16 years. He did not have any restriction of daily life at that time, but was worried about his trunk deformity.

#### Physical examination

**Fig. 1:**
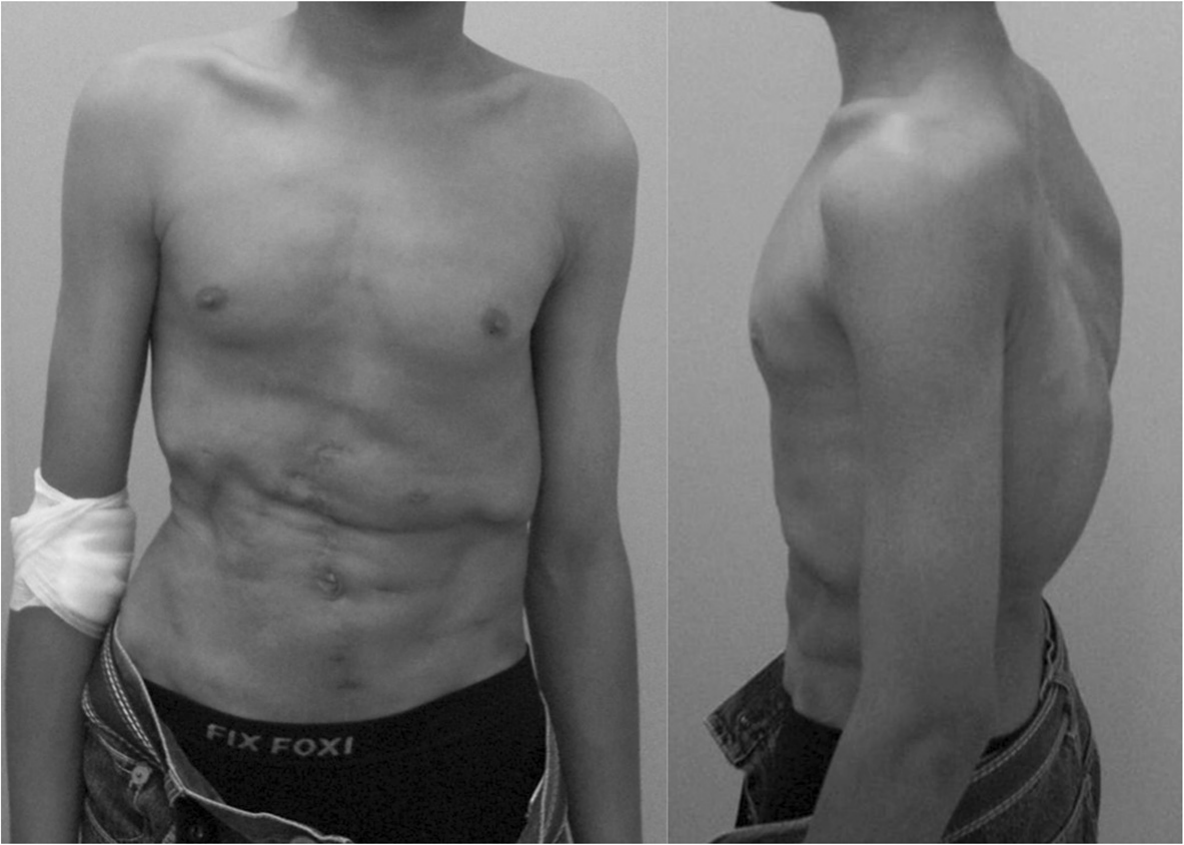
Case 1 preoperative physical appearance. Anterior view showing imbalance of shoulder and waist line. Other characteristic findings such as short stature, a small chest, and variable limb abnormalities were not found. The scar seen at the center of his abdomen was made when he received a renal transplant. Lateral view showing thoracic rib humps and thoracolumbar kyphosis

He was 160 cm (−1.9 SD), 50 kg, and thus his body mass index (BMI) was 18.5 kg/m^2^. Physical findings were imbalance of his shoulder and waist lines, a prominent right scapula and rib hump, and left lumbar hump (Fig. 1). He did not have obvious short stature or a small chest. We could not find any other abnormalities except for hypacusia. He had a scar in the center of his abdomen that was made when he received the renal transplant. The result of arterial blood gas analysis was almost normal and respiratory functional data showed moderately restrictive impairment of ventilation (vital capacity (VC) 2.25 L, %VC 54.4 %, and forced expiratory volume in 1 second (FEV1) 100 % predicted). A peripheral blood sample was obtained and all laboratory data were normal including renal function.

#### Radiographic findings

**Fig. 2:**
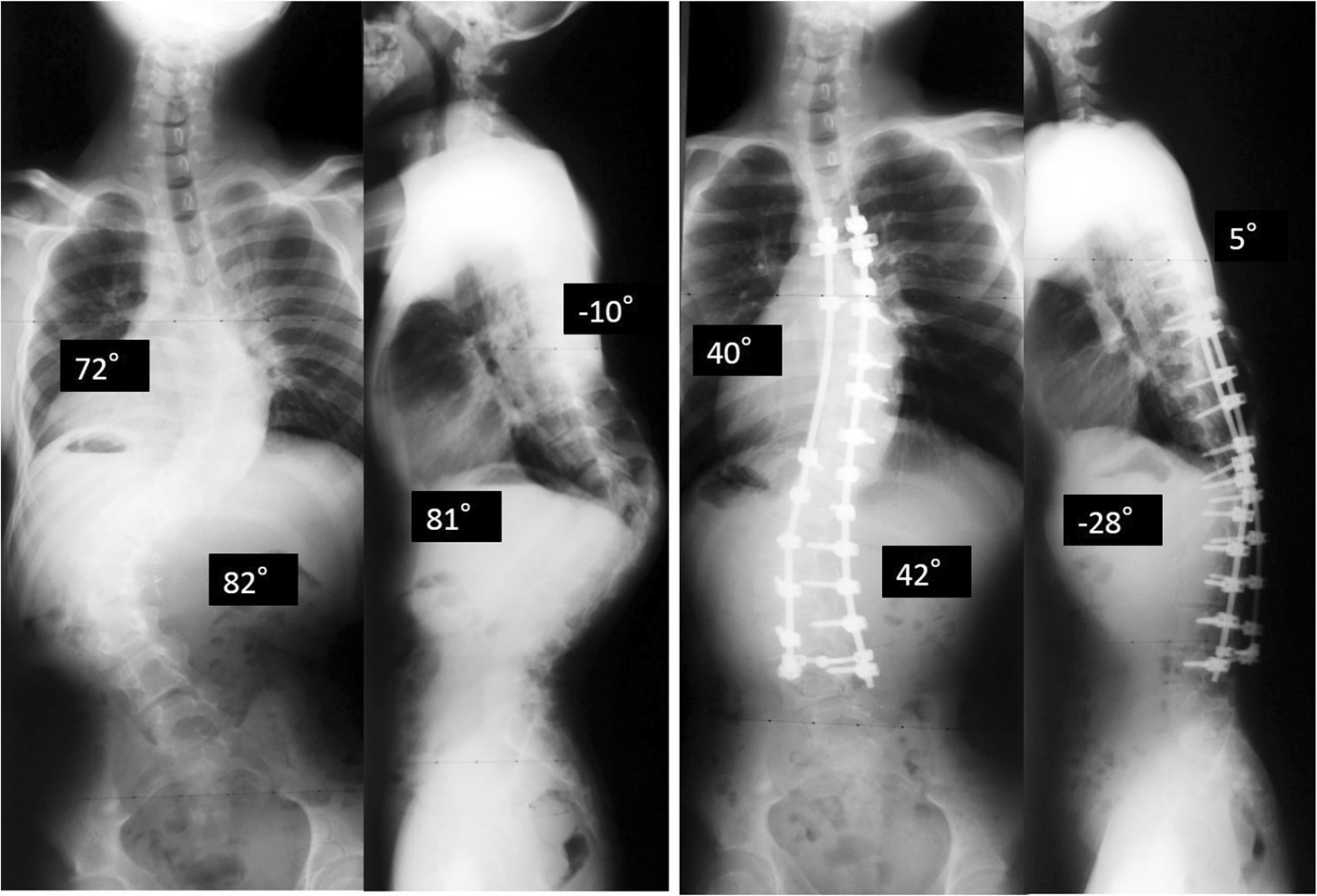
Case 1 total spine standing coronal and lateral radiographs. Coronal major curves were 70° at T4–12, 82° at T12–L3 and these curves were rigid. Sagittal total spine radiograph showed thoracic lordosis at T5–L2 was 10° and thoracolumbar kyphosis at T10–L3 was 81°. The radiographs at 2 years postoperatively showed that the major coronal Cobb angles were 40° and 42°. The thoracolumbar kyphosis between T10 and L3 decreased to 30° and was maintained

His total spine radiograph showed double major curves when we first saw him. The posterior–anterior total spine radiograph showed a coronal Cobb angle of 70° (T4–L2), 82° (T12–L3), and Risser grade V. A lateral total spine radiograph showed an 81° thoracolumbar kyphosis (T9–L3) and 10° thoracic lordosis (T5–L2) (Fig. 2). We considered that he had indications for surgical treatment. We classified his spinal deformity as Lenke type 6C [8] and planned the fusion area and surgical strategy. The risks and benefits of spinal correction were explained and informed consent was obtained from the patient and his family.

#### Surgery

Sufficient attention was paid to avoid compressing his transplanted kidney, which was placed around the right lower abdomen and to maintain sufficient flow though the kidney when he was fixed in a prone position. The fusion area was from T4 to L4, using pedicle screws, hooks, and sublaminar wirings as anchors. Ponte osteotomy was added from T12 to L2 to correct the kyphotic deformity. Before wound closing, autogenous bone grafting was performed. There were no intraoperative complications associated with respiratory dysfunction or the transplanted kidney. Cobb angles were corrected by 49 % to 36° (T4–T12) and 42° (T12–L3), respectively. Thoracolumbar kyphosis was corrected by 63 % to 30° (T9–L3).

#### Postoperative schedule

Our patient could be extubated immediately after wound closure and did not need to be treated at an intensive care unit (ICU). Oxygen supply was administered routinely for a single overnight period and respiratory dysfunction was not identified in the postoperative period. Sufficient perfusion ensured no circulatory failure of the transplanted kidney occurred. He was discharged 25 days postoperatively. His most recent radiographs at 2 years after surgery showed Cobb angles of 40° (T4–12), and 42° (T12–L3), and correction losses were 4° and 0° respectively. A lateral whole spine radiograph also showed thoracic lordosis was 5° and thoracolumbar kyphosis was 28° (Fig. 2). A respiratory function test was performed at one year after surgery, showing VC 2.47 L, %VC 55.7 %, and FEV1 95.9 %. There were no other complications, including surgical site infection, within 2 years of surgery. The patient and his family were thoroughly satisfied with his treatment, including the surgery.

### Case 2

#### History

The second patient was 24 years old at the time of surgery. She was born normally, but had a narrow, constricted thoracic cage with a poor respiratory condition, short horizontal ribs, and short limbs. At 6 months old, she was diagnosed from these physical and radiographic characteristics as having Jeune syndrome. Her respiratory condition deteriorated and she required mechanical ventilation. She required thoracoplasty at 15 months old. This case of thoracoplasty was reported by Takada et al. [9]. The surgical thoracic expansion was successfully performed and she survived her infancy. At 5 years old (about 3.5 years after thoracoplasty), her thoracic operation scar became infected and her implants were removed. At 11 years old, scoliosis was highlighted and her spinal deformity deteriorated gradually. Bracing for scoliosis for 3 years was indicated at 13 years old. At the end of her brace treatment, she was indicated for surgical correction. However, the physician hesitated to conduct surgery because of her past history of Jeune syndrome. She had matured normally and maintained a normal life, but was bothered by her trunk deformity. She took the opportunity afforded by her marriage to reconsider treatment for scoliosis. When she was 23 years old, she was referred to our hospital to consult us regarding indication for spinal correction.

#### Physical examination

**Fig. 3:**
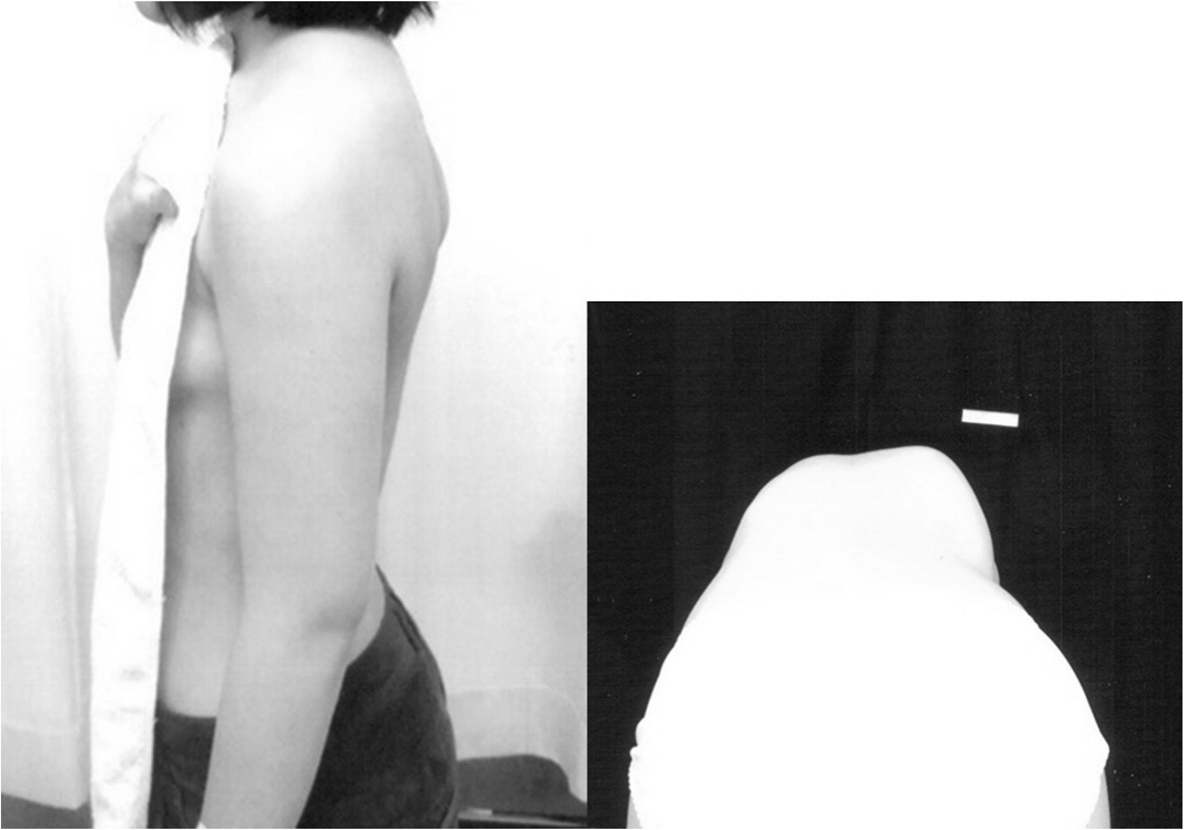
Case 2 preoperative physical appearance. Lateral view showing prominent right scapula and rib hump. However, there were no apparent abnormalities such as a small chest, obvious short stature, and short limbs. Clinical photos during forward bending showed an obvious right rib hump and mild left lumbar hump

Her height and weight were 149.7 cm (−1.6SD) and 45.3 kg, giving a BMI of 20.2 kg/m^2^. Physical findings were a prominent right scapula and rib hump, and left lumbar hump (Fig. 3). Her abnormalities, such as a small chest with horizontal ribs and short limbs, had diminished at that time. She had a scar in the center of her anterior chest wall, which was made when she received the thoracoplasty and implant removal. The result of the arterial blood gas analysis was normal and the respiratory functional data showed moderately restrictive impairment of ventilation (VC 1.78 L, %VC 56.3 %, and FEV1 89.1 %). She had no restriction in her daily life, but felt tired and listless after sitting for several hours.

#### Radiographic findings

**Fig. 4:**
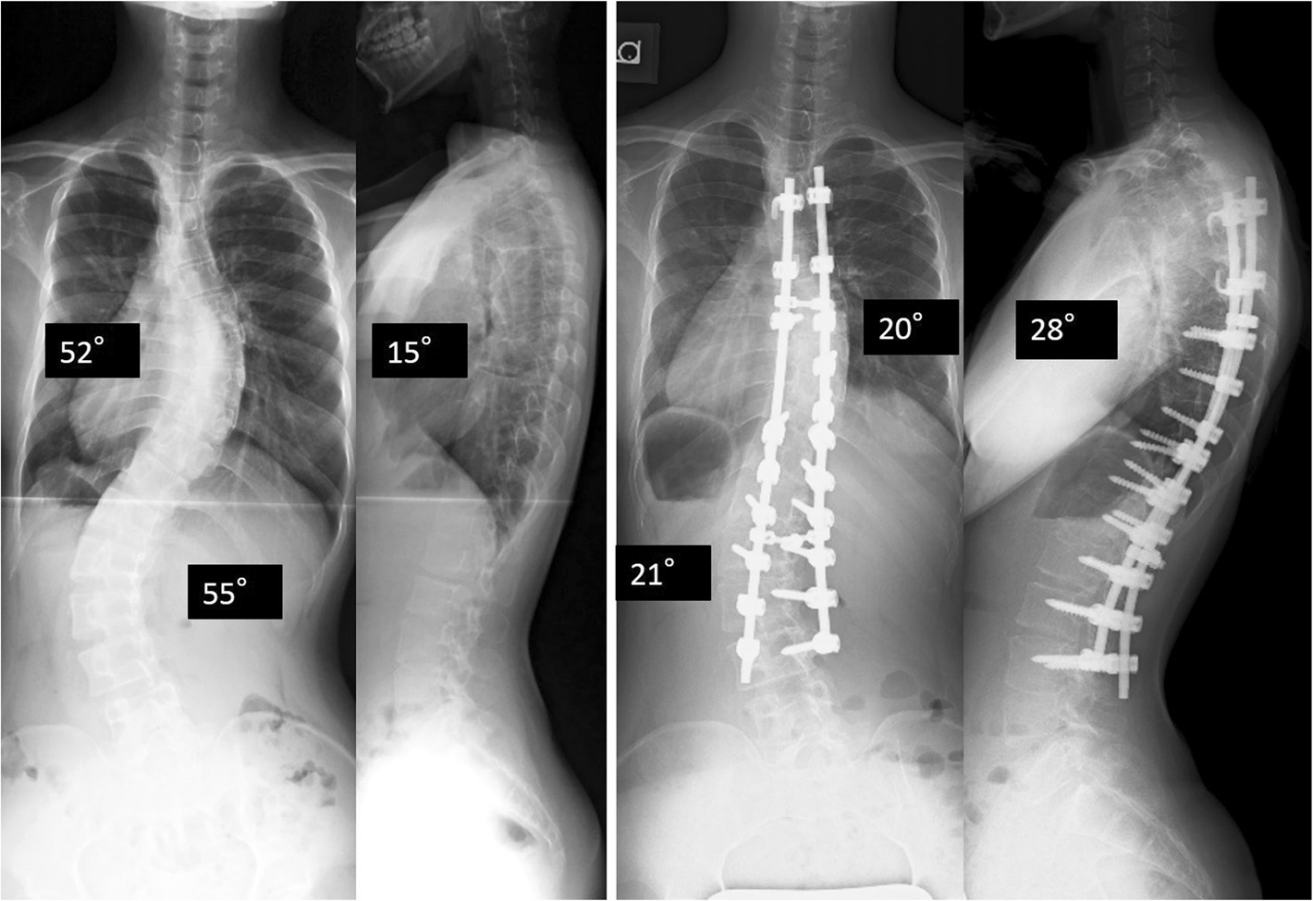
Case 2 total spine standing coronal and lateral radiographs. Preoperative coronal major curves were 52° at T5–L1, 55° at T11–L4, and thoracic kyphosis at T5–L2 was 15°. The radiographs at 2.5 years after surgery. The major coronal Cobb angle was 20° (61 % correction) and 21° (62 % correction). The sagittal Cobb angle between T5 between T12 increased to 28°

Her posterior–anterior total spinal radiograph showed a double major curve. Cobb angle was 52° (T5–11), 55° (T11–L4), and Risser grade was V (Fig. 4). Chest computed tomography showed deformity of the thoracic cavity mainly because of scoliosis and rotational deformity, but no characteristic abnormality obviously caused by Jeune syndrome. We classified her deformity as Lenke type 6CN and planned fusion levels before surgery. The risks and benefits of spinal correction were explained and informed consent was obtained from her and her family.

#### Surgery

We used a posterior approach and widely exposed posterior elements. A Ponte osteotomy was performed at several levels around thoracic and lumbar apical vertebrae. We used pedicle screws, hooks, and sublaminar tapes as anchors. To correct the deformity, we used a same procedure as we perform for AIS. Cobb angles were corrected by 61 % to 20° (T5–11) and 21° (T11–L4).

#### Postoperative schedule

Her general condition including her respiration was stable throughout the operation. She could be extubated immediately after the operation. She received oxygen for only one day after surgery. A respiratory function test showed moderately restrictive impairment of ventilation (VC 1.36 L, %VC 43.8 %, and FEV1 92.0 %) after surgery. She was discharged 13 days after surgery resumed her normal life. At 2.5 years follow up, she conducted a normal life without any restrictions and the correction of the spine was maintained (Fig. 4).

## Discussion

Here we report the cases of two patients with Jeune syndrome who survived their infancy, and underwent successful posterior spinal correction of their scoliosis after maturation.

The prevalence of Jeune syndrome is estimated to be between 1 in 100,000 and 1 in 130,000 live births [3, 10]. Diagnosis is based on clinical and radiographic findings. Clinically, patients with Jeune syndrome are characterized by a short stature, a small chest with respiratory complications, and variable limb abnormalities [3, 4]. Renal, hepatic, pancreatic, and retinal complications may also occur [1–4]. The prognosis of Jeune syndrome is associated with pulmonary and renal involvement. In infancy, most patients die because of respiratory dysfunction and renal failure is the second most prevalent cause of death. Campbell et al. [5] reported that approximately 60 to 70 % of patients die from respiratory insufficiency in their early infancy. So the indication of respiratory intensive care in early stages is considered to lead to a better survival rate [4, 11]. Renal failure occurs in up to 30 to 40 % of those patients who survive beyond the infancy and is a leading cause of death for these patients [3, 5, 12]. Successful renal transportation in this syndrome has been reported in several articles [3, 13]. Recently, vertical expandable prosthetic titanium rib (VEPTR) has been applied to manage lethal respiratory condition and/or early onset scoliosis in infantile patients with Jeune syndrome. Gadepalli et al. [14] reviewed 26 young patients including Jeune syndrome who underwent VEPTR in their early stage. They reported the usefulness of VEPTR to improve scoliosis, however, they also reported the difficulty to increase lung function and volume. O’Brien et al. [7] revealed that VEPTR improved survival rate with less ventilator dependence in Jeune syndrome. Further long-term results for VEPTER is needed in the future.

The prognosis of children with Jeune syndrome seems to be getting better because of improvement of medical care. However, Jeune syndrome has a wide clinical variation, and the prognosis for patients differs greatly [3, 5, 10]. Tüysüz et al. [12] suggested that patients could be clinically classified into three groups: the first with severe pulmonary involvement, the second with renal failure, and the third with mild prognosis. One of our two patients survived infancy, although his renal function deteriorated, which led to chronic renal dysfunction that was treated with a renal transplant at his age of 10 years. According to Tüysüz’s classification, this patient is in the renal failure group. The other patient had a narrow and constricted thoracic cage, which is typically observed in Jeune syndrome in infancy, and leads to a severe respiratory condition. She underwent thoracoplasty to treat her narrow chest cavity at 15 months. She survived her severe pulmonary condition because of this surgery. According to her history she is classified into the severe pulmonary involvement group.

We found a few reports or comments regarding the association between Jeune syndrome and spinal abnormalities. Tüysüz et al. [12] reported that one of their patients had atlantoaxial instability and spinal cord compression. Campbell et al. [5] showed 10 Jeune patients with a 60 % rate of proximal cervical stenosis at American Thoracic Society 97th International Conference, and suggested that if patients are symptomatic, then posterior decompression and spine fusion is necessary. However, in our two cases, cervical magnetic resonance imaging, which was performed before surgery, did not show any abnormality. Recently, O’Brien et al. [7] studied 24 Jeune syndrome patients and reported that scoliosis was present in 41 %, several patients required surgery. However, to our knowledge, there was hitherto no information about causes or characteristics of scoliosis in Jeune syndrome who survived their early stage. In case 1, we cannot confirm when deformity of the patient occurred or how his deformity progressed. The patient did not receive any treatment for scoliosis until he was 14 years old. If the scoliosis was related to Jeune syndrome from birth, his deformity may have required treatment at a much earlier stage. The case of spinal deformity in the second patient was highlighted for the first time when she was an adolescent. Some articles have reported that bone abnormality improved dramatically with age. Taking these things into consideration, we consider the clinical histories of spinal deformity in these two cases might be similar to those of AIS. Interestingly, we have discovered some different aspects between AIS and our cases. First, both cases were classified as Lenke type 6. The most commonly treated types in AIS are Lenke type 1 (51 %) and type 2 (20 %), the prevalence of Lenke type 6 is only 3 % [15]. Second, the patient described in Case 1 had a sharp thoracolumbar kyphosis, which is not a common deformity in AIS. Additionally, the correction rates were less in these two cases (49 % in Case 1, 62 % in Case 2) than is average for AIS (60–70 % correction) [16]. So, our limited experience suggests that although the progression of scoliosis in patients with Jeune syndrome who survived their infancy may be similar to that in AIS, the details of the type of curvature, alignment of deformed spine and rigidity of the deformity might be different from that in AIS.

When scoliosis correction is indicated in Jeune syndrome patients the respiratory condition of the patient is a large concern. Respiratory condition may improve with age. Keppler-Noreuil et al. [3] reported that the thoracic malformation and the respiratory problems may improve with age. Amirou et al. [13] reported a patient whose thoracic deformity had disappeared when they underwent renal transplantation. Our two patients had no restriction in their daily life and we could not find any characteristics of Jeune syndrome in their preoperative chest radiographs. Preoperative respiratory function tests showed moderate restrictive respiratory dysfunction in both cases, which were of similar severity to that seen in AIS. Although the anesthesiologists closely evaluated respiratory conditions throughout the surgical procedure and carefully judged their condition before extubation, they did not use extraordinary anesthetic techniques in the surgical treatment of these patients.

## Conclusions

Spinal correction surgery can be safely performed in patients with Jeune syndrome using a posterior approach following similar strategies as used for AIS. The patient described in Case 2 most likely missed the most optimal timing for surgical intervention. The experience of these two cases suggests that, for Jeune syndrome patients who have survived infancy, surgical intervention is indicated if it can be performed safely. We highlight that the timing of this surgical intervention is important, especially for patients diagnosed with Jeune syndrome, and we should not hesitate to plan surgery where multiple risks for anesthesia (e.g., pulmonary or renal dysfunction) are tolerable, otherwise spinal deformity will progress and correction cannot be performed properly.

## Consent

Written informed consent was obtained from the patients for the publication of their cases and any accompanying images. A copy of the written consent is available for review from the Editor-in-Chief of this journal.
